# Cultural barriers to male partners’ involvement in antenatal care in Limpopo province

**DOI:** 10.4102/hsag.v29i0.2322

**Published:** 2024-01-31

**Authors:** Kenneth V. Nesane, Fhumulani M. Mulaudzi

**Affiliations:** 1 Department of Nursing Science, Faculty of Health Sciences, University of Pretoria, Tshwane, South Africa

**Keywords:** antenatal care, culture, male partner, cultural male involvement, cultural barriers

## Abstract

**Background:**

Participation of male partners in antenatal care (ANC) is a complicated process that involves social and behavioural transformation. It necessitates that males take a more active part in reproductive health. Men’s participation in prenatal care has been linked to beneficial health outcomes such as enhanced maternal health outcomes across the world. However, culture has been identified as a barrier to male partners’ participation in prenatal care.

**Aim:**

The aim of the study was to explore and describe the cultural barriers to male partner involvement in ANC.

**Setting:**

The study focussed on selected clinics and hospitals under Vhembe District, Limpopo province.

**Methods:**

Qualitative, exploratory, descriptive, and contextual research design was used in this study. Qualitative data were collected through individual semi-structured interviews and Focus Group Discussions (FGDs). A thematic analysis approach was used to analyse the collected data from semi-structured interviews and FGDs.

**Results:**

The findings revealed three themes: cultural beliefs and practices that affect male partners’ involvement in ANC; gender-related barriers that affect male partners’ involvement in ANC; and socioeconomic barriers to male partners’ involvement in ANC.

**Conclusion:**

The study’s findings revealed that certain cultural beliefs and practices are a stumbling block to male partners’ involvement in antenatal healthcare.

**Contribution:**

Culturally based developed strategy might help in improving the knowledge and practices of male partners in ANC.

## Introduction

Globally, antenatal care (ANC) is an important part of women’s pregnancy journey and is viewed as their domain. By attending antenatal care, women get the opportunity to identify and address underlying illnesses and early signs of pregnancy complications (Davis et al. 2018). Antenatal care appears to increase the usage of emergency obstetric care and motivate women to deliver at a public health facility (Påfs et al. 2015). However, the male partners are typically not included in ANC despite that they have played a crucial role from the conception through to the delivery of the child (Morgan et al. 2022). Several impediments have been recognised by researchers as barriers to male engagement; and culture is viewed as a significant relevant element. Culture is the biggest impediment to male partners’ engagement in ANC in many countries across the world (Ongolly & Bukachi 2019). In several African faiths, it is considered a taboo and highly unnatural. Therefore, in South African rural areas, some male partners do not participate in ANC, claiming taboo as a barrier (Falade-Fatila & Adebayo 2020).

The variance between culture and beliefs in other countries is that an African man is raised with the mentality that the responsibility of ANC is only of the women (Falade-Fatila et al. 2020). Furthermore, South African men assume that ANC is a women’s organisation. Because male partners are viewed as decision makers in ANC, they must adhere to cultural norms when it comes to maternal health concerns (Ongolly et al. 2019). However, in Tanzania, socioeconomic, cultural, religious and ethnic disparities continue to inhibit women’s ability to make decisions regarding ANC, because of men’s allocation of family income, transportation and time, and access to health services (Mapunda et al. 2022). In other cases, women do not have a say in cultural matters. Therefore, it also relies on the individual’s educational standing (Bayeh 2016). In Madagascar, the majority of male partners reside in remote rural regions where they continue to practise traditional norms that prevent men from engaging in ANC (Morris et al. 2014).

In many African cultures, pregnant women are well respected, and pregnancy is viewed as a blessing from God (Ongolly et al. 2019). Similarly, in the Vhavenda culture, a pregnant woman is considered an important person, and pregnancy is treated with respect. It is a customary practice in South Africa that pregnant women are not permitted to remain in a queue for public services unless they are at the clinic where they are seeking comparable services. In Kenya, healthcare providers encourage male partners to be involved in ANC (Ongolly et al. 2019).

Despite a dearth of male engagement in some situations, male partners’ involvement in ANC has improved across Europe as a result of government-imposed efforts to increase male partners’ involvement in ANC (Maluka & Peneza 2018). Furthermore, in the United Kingdom, male partners participate in ANC because they feel that a man must work to support his family (Mathews, MacDorman & Thoma 2015). Another option for increasing pregnant women’s access to critical healthcare, such as ANC, is to enhance male partners’ participation in maternal health services (Yaya et al. 2019). Several reasons are attributed to this; no other men are available in the waiting rooms, negative healthcare provider treatment of male partners, and long waiting times (Yende et al. 2017). Therefore, there is a need to explore and describe cultural barriers and practices affecting male partners’ involvement in ANC and address problems related to the barriers and enablers that hinder male partners’ involvement in ANC. Therefore, the purpose of this study was to explore and describe cultural barriers and enablers that affect male partners’ involvement in ANC.

### Problem statement

Worldwide, men are considered key decision-makers and chief providers, often determining women’s access to economic resources. Furthermore, male partners have a significant impact on the availability of healthy food, women’s workload, and the distribution of money, transportation, and time for women to visit health facilities (Matseke et al. 2017). As a result, World Health Organization (WHO) recommends that male partners be included in ANC concerns (WHO 2015). They should get a complete education about minor pregnancy problems, nutrition, desires and vomiting during pregnancy, and other expectations during ANC visits with their partners to counsel and assist the women (Kiptoo & Kipmerewo 2017).

However, in South Africa, some males are not active in ANC services, and they are unaware of what is truly required of them during ANC (Nesane, Maputle & Shilubane 2016). The absence of male partners’ engagement in ANC is mostly because of their cultural views that forbid men from engaging in ANC (Younas et al. 2020). The continued lack of involvement of men in ANC services could lead to few women seeking maternal health services, and as a result, worsening the negative maternal health outcomes for women (Selection, Medicines & Organization 2014; Mersha 2018). Men are not involved and they appear to be comfortable because they are not involved in ANC and they may not realise when pregnant women require medical treatment. As a result, women postpone getting medical treatment. This lag might increase maternal morbidity and death (Matseke et al. 2017). This study intends to investigate the cultural barriers and facilitators influencing male partners’ engagement in ANC to make recommendations that would increase men’s involvement in ANC.

## Research methods and design

### Study design

A qualitative method using an explorative, descriptive, and contextual research design was undertaken (Rutberg & Bouikidis 2018). The contextual design allowed the researcher to explore cultural barriers to male partners’ involvement in ANC.

### Research setting

This study took place at the public health facilities, which include 10 clinics and 7 hospitals which were selected in the Vhembe district, Limpopo province, South Africa. The clinics were selected because of the high number of deliveries per facility, and all hospitals in the Vhembe district were included.

### Population and sampling

The study population consisted of males who accompanied their pregnant partners and males partners who came for ANC consultation at the selected government clinics and hospitals of the Vhembe district. Male partners usually stay in the cars while accompanying their partners at the health facilities. The male partners who were included were those who agreed to participate. A convenience sampling method was used. This sampling method was carefully selected to diversify and obtain rich information from the participants. Ten clinics were selected because of the highest number of ANC attended there per month. All the seven hospitals in the Vhembe district were selected as they are the referral hospitals for the clinics. Only male partners aged 18 years and above were included in this study. Those who agreed to participate were given a further explanation of the study and a consent form to sign as participation in the study was voluntary (Guetterman 2015).

### Data collection procedures

Qualitative data were collected through individual semi-structured interviews and focus group discussions (FGDs). The FGDs were facilitated by the researcher. Individual in-depth interviews were carried out with 22 individuals, and data saturation was achieved after interviewing 16 of them. Each FGD had a total of seven participants. The researcher arrived at the clinic early in the morning, waited for the male partners who accompanied their partners for ANC and those who came for consultation, then addressed them, described the study to them, and asked if they were interested to participate. Only four male partners from the participants were at the health facility accompanying their partners. Interviews lasted between 30 min and 45 min per session. As a result of coronavirus disease 2019 (COVID-19) requirements, interviews were completed in one of the facility’s counselling rooms and over the phone. Participants were informed that their participation was voluntary and that the information they provided will be managed in a confidential manner by not revealing their names during reporting on, or publication of the study. Some individuals were contacted at clinics and hospitals and data collection was facilitated over the phone. Interviews took place between November 2021 and March 2022. Each facility was visited twice.

As some of the interviews were conducted over the phone, the researcher took notes and utilised probing to facilitate a deeper investigation of the event. The researcher gathered data in English. A semi-structured interview guide was created by the researcher. The researcher produced a list of pre-determined questions to be asked of the participants during the interview. After accepting to take part in the study, the participants completed the permission form (Krueger & Casey 2015).

### Data analysis

Data analysis continued concurrently with data collection because the researcher reflected on the raw data as these became available. All the recorded interviews were transcribed verbatim. A thematic analysis approach was used to analyse the collected data from semi-structured interviews (Maguire & Delahunt 2017). Thematic analysis is the process of coding qualitative data to generate themes. The thematic analysis enables the researcher to detect patterns in the data and then build themes or typologies that define these patterns. Thematic analysis (Guest, MacQueen & Namey 2011) was employed. To increase familiarity with the data, the researcher transcribed the interview recordings. There are six processes of performing thematic analysis: firstly; familiarisation, secondly; creating initial codes, thirdly; looking for themes, fourthly; evaluating themes, fifthly; defining and labelling themes, and sixthly; producing the report.

To accomplish familiarisation, verbatim transcripts were checked for accuracy by conducting a thorough reading and re-reading of each interview transcript while listening to the audio recording. An independent coder, who was an expert in qualitative research, was given the data for analysis, and a consensus discussion was held to discuss the final themes. After the researcher had completed the coding process and identified the emerging themes, categories and subcategories from the data, the researcher scheduled a meeting with the independent co-coder to discuss the findings. Atlas TI software was used during coding. Open coding was used during data analysis. The independent co-coder’s inputs were incorporated to refine the coding process. In this study, the data were analysed until saturation of data and consensus of the themes, categories and subcategories was reached between the researcher and the independent co-coder. A literature control was used to verify the findings.

### Trustworthiness

The model of Lincoln and Guba (1985) as described by Babbie and Mouton (2002), De Vos et al. (2005) was utilised to ensure trustworthiness, credibility and transferability, confirmability and dependability and authenticity of the findings. Transferability was ensured through data saturation and a dense description of the study design and methods, and the findings were made available with supporting quotes from participants. Dependability was aided by literature control, extended engagement, and member checking, and confirmability was provided by data triangulation. To avoid influencing the participants’ replies, the researcher practised flexibility by writing down his own background and experience. The data acquired through the interview guide was checked by an independent coder in this study.

### Ethical considerations

Ethical clearance to conduct this study was obtained from the University of Pretoria Research Ethics Committee (No. 261/2021).

The researcher also obtained permission to collect data in the health facilities from the Limpopo province Department of Health. The nursing service managers of the chosen facilities were contacted and asked to undertake the research. Throughout the procedure, the researcher ensured that the participant’s right to privacy, anonymity and secrecy, fair treatment, and protection from discomfort and injury were all taken into account. Before the interviews, anonymous individuals provided written informed permission. Participation in this study was fully voluntary, and participants were free to leave at any moment without penalty. Only the researcher, an independent coder, and the two study supervisors had access to the collected data. To preserve their identity and privacy, participants were given a number that was utilised throughout the data-gathering procedure.

## Results

### Demographic profile of participants

Participants were 22 male partners between the age of 18 and 55 years. A total of 14 male partners worked, while 8 did not. Furthermore, 16 of them were Christians while 6 believed in African culture. There were 17 from Vhavenda culture while 5 were from Tsonga culture. A total of 8 had Bachelor’s degrees, 7 had diplomas and the other 7 had matric.

### Themes

Three themes and eight subcategories emerged from data analysis. Table 1 presents the themes and categories.

**TABLE 1:** Themes and categories.

Themes	Subcategories
Cultural beliefs and practices that affect male partners’ involvement in ANC	Cultural beliefs regulating male involvement in ANC Cultural beliefs affecting pregnant women Prevention of harm to men Prevention of harm to women
Gender-related barriers affecting male partners’ involvement in ANC	Gender-related barriers (segregating male and female responsibilities)
Socioeconomic barriers	Work and finance related barriers Health-related barriers Information-related barriers

ANC, antenatal care.

#### Theme 1: Cultural beliefs and practices that affect male partners’ involvement in antenatal care

From the interviews conducted, it was discovered that in the Vhavenda and Vatsonga cultures, male partners were not allowed to take part in ANC. Pregnancy has been viewed as a women’s domain for a long time. Many women suffered during their pregnancies as a result of their spouses’ failure to provide the necessary assistance. The selected categories under this issue include cultural beliefs governing male engagement in ANC, cultural beliefs impacting pregnant women, prevention of harm to women, and prevention of harm to women.

**Cultural beliefs regulating male involvement in antenatal care:** Participants expressed some cultural beliefs that regulated them from being involved in ANC. These cultural beliefs include: norms, traditions, and cultural views that pregnancy is a women’s domain. This is supported by the following quotations:

‘In the Venda tradition, when we were growing up, it was not allowed for a man to become involved in ANC or any pregnancy-related activities. Traditionally, only elderly women were allowed to interact and be involved in the advisory role.’ (P1, 26 years old, male)

‘Because I have a bit of knowledge about my culture (traditional times) when a woman is pregnant and her days are due, I take her home to stay with her mother and give birth while there being taken care of by her mother. She returns when the child is four–six months old.’ (P3, 31 years old, male)

‘Yes, our culture influences us. I am a Venda man, so culturally, many things are not allowed to be done by men when the wife is pregnant; we are expected not to be much involved because when the wife is pregnant, she is a responsibility of the older people of the family not to the younger people or the husband.’ (P5, 38 years old, male)

**Cultural beliefs affecting pregnant women:** Some participants expressed that the Vhavenda culture hinders male partners’ involvement in ANC, with some cultural beliefs negatively affecting pregnant women and becoming a barrier to attaining quality ANC. This is supported by the following quotations:

‘There are certain cultural beliefs that affect the well-being of a pregnant woman for example the tradition does not allow a pregnant woman to travel. It is a problem when I want to take my wife to the city.’ (P1, 26 years old, male)

‘Yes sometimes some cultural beliefs prohibit male partners from being involved in antenatal care. Things like, if you accompany you wife to the clinic you should not tell on the way because it will delay your partner during labour.’ (P4, 44 years old, male)

**Prevention of harm to men:** Prevention of harm to men was considered an important matter in Vhavenda culture, which consequently prohibited male partners from being involved in ANC. Participants revealed that there is a view that if men see and know what takes place in the ANC consulting rooms and during delivery, they may be affected psychologically and lose interest in sex. This is supported by the following quotations:

‘Eish! The older people are protecting us from harm, and if I see how difficult it is for women at the clinic during examination and childbirth, I will lose interest in having sex.’ (P11, 44 years old, male)

‘I am afraid of going inside and seeing it. I think I will no longer be free to be with my wife again if I see what is happening during ANC consultations and childbirth.’ (P10, 18 years old, male)

‘They say that if male partner enters the consultation room during antenatal care, male partner can lose interest to women.’ (P6, 38 years old, male)

**Prevention of harm to women:** Some participants expressed that in their culture when their partners are pregnant, they are expected to limit sexual activities with them so that the men cannot bring home sexually transmitted diseases and stated:

‘Culturally, it is not allowed to have sex when the woman is pregnant; this is to prevent sexually transmitted diseases to pregnant women.’ (P9, 24 years old, male)

‘Old people always assume that male partner Sir [*sic*] another partners outside the relationship. That’s why they say we should not have too much sex when the wife is pregnant.’ (P7, 40 years old, male)

During the FGDs, the participants emphasised the issue of men having multiple partners during pregnancy as a challenge. It was expressed that because men are prohibited from having sex with their pregnant wives, some male partners find other women who will satisfy their sexual desires for that period. This is expressed as follows:

‘We are discouraged to sleep with our wives because they know that men will always be men. We will be going out with extramarital sex, so they do not want us to bring back the diseases from other women, so they are trying to protect this woman from sexually transmitted diseases and other diseases.’ (P4, 44 years old, male)

‘We are not allowed to touch her or come close to her. We did not have to trouble her during the pregnancy. We are also not allowed to upset her, that’s why we also try not to let her see text messages from other ladies as she will become jealous.’ (P2, 49 years old, male)

#### Theme 2: Gender-related barriers that affect male partners’ involvement in antenatal care

Gender-related barriers seemed to influence male partners to refrain from attending ANC, as they viewed pregnancy as a women’s domain. Some participants revealed that pregnancy is for women and men have to work and provide for the family. Gender-related barriers that affected male partners’ involvement in ANC included segregating male and female responsibilities.

**Gender-related barriers (segregating male and female responsibilities):** Participants viewed gender-related barriers that involved the segregation of male and female responsibilities to influence the thinking that it is the pregnant woman’s responsibility to go to the clinic or hospital for antenatal visits. Male partners revealed that they are shy to enter government clinics and hospitals because these healthcare facilities are always full of women seeking medical care:

‘No, I can’t go to the clinic because I’m a shy man. I cannot be there in the clinic with a lot of ladies there in the clinic so for me, I think I cannot be there, and I am not supposed to be there.’ (P5, 38 years old, male)

‘Going inside the clinic is for this young man. I am old and I cannot go inside the clinic which is full of women.’ (P11, 48 years old, male)

Similarly, one participant stated the following:

‘I think ANC is a women’s issue. What will I do at the clinic? What will people think of me.’ (P2, 48 years old, male)

‘I normally don’t enter the clinic with my wife when she’s pregnant. That clinic is always full of ladies.’ (P8, 42 years old, male)

#### Theme 3: Socioeconomic barriers

Socio-economic status was viewed as a barrier to some male partners’ involvement in ANC. As the majority of the men were unemployed, this made it difficult for them to accompany their wives to the healthcare facilities to attend ANC. Instead of going with their partner, some male partners would opt to give money to the wife so that they can save as the healthcare facilities are too far. The socio-economic barriers included work and finance related barriers, healthcare system-related barriers, and information-related barriers.

**Work and finance related barriers:** Participants revealed that as male partners, they are involved in ANC financially. They provide for their families and make sure that their wives get everything they want during pregnancy. This is supported by the following quotations:

‘With me, I make sure that my wife is having money so that if anything happened related to the pregnancy, she will be able to consult as early as possible to avoid a lot of complications due to lack of money.’ (P16, 37 years old, male)

‘Uh yes, I do accompany her but not most of the time because there are transport constraints and the clinic is far so I can’t spend much money because I don’t have that money because to go to the clinic is around R25 each person so to come back is R50 which is R100 and so if she can go alone at least we can be able to save at least that the future and we can buy something.’ (P2, 48 years old, male)

‘The clinic is very far from here. We pay a lot from here to a clinic. It is better for her to go alone so that we can save money.’ (P19, 51 years old, male)

**Healthcare system-related barriers:** Participants expressed that they felt that the public hospitals and clinics were not built in such a way that male partners could enter the consultation room with their partners without invading other patients’ privacy; therefore, it hindered male partners’ involvement in ANC. The male partners also indicated that hospitals were not designed to accommodate males or anyone who accompany a pregnant woman. The system and the environment were also not conducive for males to feel free to be able to support their partners:

‘In a public hospital, it’s a big room with so many beds, because there are other people who also want to give birth, so men are not allowed to enter.’ (P3, 31 years old, male)

‘There is also no space for males at the ANC clinic. It is often overcrowded, and you feel out of place as a man.’ (P12, 44 years old, male)

**Information-related barriers:** Some male participants expressed a lack of information and knowledge about ANC. They also revealed that it is important for male partners to have information related to ANC so that they can be involved as early as possible to save the baby and the mother’s lives. This is supported by the following quotations:

‘Not at all because even if you can go anywhere, you won’t find the pamphlets or the notice saying men should be involved in pregnancy or something to show that men are important during pregnancy. what you see are only children and women advert.’ (P2, 48 years old, male)

‘I hardly see anything related to antenatal care here and the clinic and even when I visit the hospital I have never seen anything talking about antenatal care.’ (P21, 49 years old, male)

## Discussion of findings

The findings revealed barriers to male partners ‘involvement in ANC. Male partners were controlled by culture in their daily lives. According to the interviewees, certain (various) cultural ideas and behaviours still prevent males from participating in ANC. Other male partners expressed enthusiasm about participating in ANC, while others did not explain their absence. Male participation has generally been portrayed as either obstructive, limiting women’s decision-making on the use of family planning, or non-existent, with male partners absence entirely owing to a lack of interest in reproductive health issues. However, in many historically patriarchal cultures, males dominate decision-making over family size and ANC (Kabagenyi et al. 2014).

Cultural barriers take the lead in preventing and encouraging male partners’ involvement in ANC. As culture is the main barrier to male partners’ involvement in ANC, other male partners, especially youth, ignore culture and support their partners during ANC; hence, older male partners cited culture as the stumbling block to involvement in ANC.

Cultural beliefs regulating male partners’ involvement in ANC seem to be among the stumbling blocks of their involvement in ANC. Male partners follow cultural traditions that prevent them from being involved in ANC. Most developing nations have patriarchal systems, with males serving as the major decision-maker and managers of the home finances. As a result, the male partner or spouse generally chooses to seek care (the initial delay). However, male companions are not expected to be directly involved in their wife’s pregnancy and delivery care in other cultures. If they are, their peers will perceive this as a sign of weakness (Suandi, Williams & Bhattacharya 2020). According to the findings of this study, some male partners were hesitant to participate in ANC because they did not want to be perceived as weak men. Furthermore, male partners assume that a man must be strong and take on all of the responsibility.

In this study, several male partners were somewhat involved in prenatal care, indicating that culture limits their involvement. However, in sub-Saharan Africa, the participation of male sexual partners in maternity care is a contentious and complex subject; historically, males do not accompany their wives to maternity clinics and pregnancy is considered a woman’s affair (Jefferys et al. 2015). This is different from some of the male partners in the study, as they participated while their partners were pregnant.

From the interviews conducted in this study, male partners viewed involvement in ANC as a difficult thing to do, as the woman is the one who should attend antenatal clinics. Similarly, male companions are uncommon in prenatal clinics in sub-Saharan Africa. Men coming to ANC check-ups with their partners may be inconceivable in some areas (Babalola & Fatusi 2009). Meanwhile, within that socio-cultural environment, males have absolute control in the household in terms of decision-making (Story et al. 2012). Because men determine the majority of family decisions, some experts think that male partner engagement in ANC is critical in resolving the first two of the three delays that contribute to maternal death (Kumbeni et al. 2019). In this study, male partners preferred private health facilities over public health facilities because private facilities are not full and have enough space to accommodate male partners.

In this study, some male partners wanted to participate in ANC, but they were shy, and they thought about what other people will say when they see them supporting the pregnant woman at the health facility. Cultural beliefs regulating male partners’ involvement in ANC are barriers to their involvement in ANC. Male partners grew up traditionally and they prefer to follow their traditions. During the interviews, the researcher found out that there are cultural beliefs that regulate pregnant women. In other cultures, pregnant women are not allowed to do as they wish. In this study, the researcher found out that there are women who are regulated by cultural traditions. Furthermore, they (pregnant women) decide on their pregnancy concerning what they believe in.

Whereas, Mapunda et al. (2022) found that socioeconomic, cultural, religious and ethnic disparities continue to inhibit women’s ability to make decisions regarding their health because of men’s control of the allocation of family income, transportation and time, and access to health services. Male involvement in maternal health services remains a challenge to safe motherhood, despite its essential role in providing financial, emotional, and physical support to women. Efforts to engage male partners in maternity care not only prevent delays in receiving appropriate care but also facilitate adequate treatment at the appropriately equipped health facility level (Mapunda et al. 2022). Some other efforts to engage men in ANC are hindered by cultural beliefs that regulate pregnant women. Cultural beliefs seem to be a stumbling block to male partners’ involvement in the ANC.

The Vhavenda tradition prohibits male partners from being involved in ANC. The tradition itself does not allow male partners to take part when the woman is pregnant, because they believe that the male partner will lose interest in the woman. From a social perspective, the notion of joining one’s wife at an antenatal clinic is unusual in many communities, and the husband’s presence is often considered superfluous. Perceived traditional gender roles and a lack of knowledge and opportunities for involvement in obstetric care have been reported as barriers to male partner involvement in rural Tanzania (Suandi et al. 2020).

The findings of the study revealed that healthcare providers encourage men to actively participate in ANC, partly because in most cultures, men influence most decisions made within their families, including those concerning the health of their family members. Decisions regarding when, where, and how women and children access healthcare are often made by men. This is influenced by men’s position as providers and decision makers in their households (Ongolly & Bukachi 2019). Other cultural beliefs are there to prevent harm to male partners. Male partners follow cultural traditions to protect themselves from harm.

During the interviews, the researcher observed that cultural ideas govern male partners’ participation in ANC to protect women. These cultural beliefs protect pregnant women and elevate them to a higher status during their pregnancy. Similarly, in Ghana, where patriarchal norms such as men’s control over household decision-making and greater mobility in public spaces are prevalent, it has been argued that maternal and child survival necessitates active participation of men, as well as improvements in comprehensive and basic obstetric and child healthcare coverage and quality (Kwambai et al. 2013). In patrilineal South Asia, the role of men in maternal healthcare has been acknowledged (Thapa & Niehof 2013). Similarly, in South Africa male partners’ involvement is recognised because men are given 10 paternity leave days after the delivery of the baby. Indeed, male involvement is a key component of the recent WHO’s (2015) recommendations on health promotion interventions for maternal and newborn health (Ganle et al. 2016). There are cultural beliefs that protect or prevent harm to women. Male partners seem to be following them hence low involvement in ANC.

The researcher discovered that male partners viewed the ANC as a woman’s domain and they did not want to be involved in it. Segregating male and female responsibilities is a barrier to male partners’ involvement in ANC because male partners would not want to do the duties of women. However, male partners are rarely seen in healthcare facilities and have little contact with healthcare workers, which limits their exposure to health information. Older women, especially the male partner’s mother, are regarded as experts on infant care and feeding (Forbes et al. 2018).

Men’s involvement in reproductive health and their behaviour imposes a positive impact on women’s reproductive health and the well-being of the children and society as well. The involvement of males in reproductive health has recently been promoted as a promising new strategy to improve maternal and child health (Asefa, Geleto & Dessie 2014).

Men are an important stakeholder and should be considered as half of the equation in maternal and child health. Even though men have important decision-making roles related to maternal and child health issues, in many sub-Saharan African countries including Ethiopia, maternal and child health is viewed as a woman’s affair (Mohammed et al. 2019). The lack of participation of men in antenatal, postnatal, newborn, or post-abortion care may be because they do not benefit from any information provided by health providers regarding the health of the mother and the baby or their roles in it (Mohammed et al. 2019).

Societal ascriptions of gender roles for men and women strongly influence access to skilled birth care for pregnant women. The gender division of household roles usually makes male partners heads of their households. Being the heads of their households, the male partners usually decide when and what means of accessing healthcare the family including the women should use. Male partner involvement refers to ‘the various ways in which men relate to reproductive health problems and programmes, reproductive rights, and reproductive behaviour’. Male partner involvement in maternal healthcare also refers to the direct assistance provided by men to improve their partners’ and children’s health through the perinatal, antenatal, labour, and delivery period (Saah et al. 2019). Culturally, there are duties that are performed by men and duties that are performed by women. Antenatal care is viewed as women’s domain and men do not see the importance of being involved.

During the interviews, the researcher discovered that male partners lacked information about their involvement in ANC. If male partners were provided with information, they would have been involved in ANC. Globally, the big challenge is to improve maternal health and male attendance at skilled ANC and delivery care facilities. In sub-Saharan Africa, the absence of support from the husband is one of the main reasons for many pregnant women not seeking maternity services. This indicates that the characteristics of the health delivery system, economic and geographic accessibility, and socio-demographic factors are not the only factors affecting the uptake of maternal healthcare (MHC) (Tessema et al. 2021).

It was reported that many men in low-resource countries including South Africa do not accompany their partners to the health facility during pregnancy unless there is a complication. Others wait outside the clinic, while the woman participates in health talk and consultation with the healthcare provider. Therefore, men are often unaware of the health promotion and disease prevention strategies discussed in these sessions. Men often control the family finance; thus, they may disregard health promotion until complications arise partly because of ignorance (Adeniran et al. 2015). The majority of male partners lack information about ANC. A lack of information about their role in ANC is a barrier to male partners’ involvement in ANC.

Male partners verbalised that they work far from home, and it is difficult for them to be available during ANC. Some other male partners indicated that the clinics and hospitals are situated far apart; hence, it will cost them to accompany their partners to seek medical care. Other barriers include transportation problems and a lack of social and financial support from a family member. Women are socially expected to ask permission from their male partners before making decisions about their healthcare utilisation. Besides the husband, other key actors in the referral of pregnant women to health services in southern Mozambique include matrons (influential older women), community health workers (CHWs), and neighbours (Galle et al. 2019).

Male partners always give a reason that they cannot be involved in ANC because they are always at work or work far from home. Some give some reasons that they are situated far from clinics, and the transport is expensive, especially when there are two fares.

The health system does not favour male partners’ involvement in the ANC. If male partners opted to be with their wives in the consultation room, it means they would be invading other patients’ privacy which is not allowed.

In sub-Saharan Africa, however, men were considered a major obstacle to women’s use of maternal health services. For example, men often restricted women’s access to resources necessary for safe delivery and were generally viewed by health promoters as uncaring and negligent. Reproductive health programmes typically focused on empowering women to make decisions about their healthcare and paid little attention to the prevailing gender dynamics in the household, where men were the primary decision-makers. In the 1990s, greater recognition of gender and power dynamics led the international development community to acknowledge men as potentially useful contributors to sexual and reproductive health promotion (Story et al. 2016).

The study also revealed that male partners do not like queuing in public hospitals or clinics. As a result, male partners prefer to wait for their partners outside the clinics and hospitals.

### Strengths and limitation

The study provided a broader understanding and insight into how a male partner’s involvement in ANC can assist pregnant women. In addition, the use of an explorative design enabled participants to freely narrate and interpret their experiences and views and make suggestions for improvements. Furthermore, the use of convenience sampling allowed the researcher to select the sample based on the participant’s knowledge of the phenomena being studied, which helped the researcher to obtain in-depth information and prevent bias. The results obtained in this study were collected from health facilities in Vhembe District. Consequently, it cannot be said that the findings of this study are representative of all the districts.

### Recommendations

The following culturally based recommendations were suggested to improve male partners’ involvement in ANC.

The Department of Health should have many awareness programs and campaigns to improve male involvement in ANC.

The education curriculum of healthcare practitioners (nurses and doctors) should include the collaboration of culture and modern practices during pregnancy so that healthcare practitioners can interact with male partners and educate them on their roles while the partner is pregnant.

Policy guidelines should be developed to assist in the development and creativity of time and space allocation for male involvement in ANC. There must be appropriate recommendations that all male partners who accompany their partners for ANC and delivery are not turned away after visiting hours. They must be allowed to be with their partners during ANC.

The same research topic can be repeated on a wider scale in the province to develop a model of male partners’ involvement in MHC services.

## Conclusion

Culture seems to be the main barrier to male partners’ involvement in ANC. Some of the male partners are partially involved in ANC while others are not involved. Most of the participants who viewed male partners’ involvement in antenatal as women’s domain, are men between the age of 50 and 60 years. Some young male partners seem to be eager to be involved in ANC, the challenge is that they do not know their roles in ANC. Male partners do not like to utilise public health facilities because of long queues and long waiting time. Even the structures of public health facilities do not accommodate male involvement in ANC.

## References

[CIT0001] Adeniran, A.S., Aboyeji, A.P., Fawole, A.A., Balogun, O.R., Adesina, K.T., Adeniran, P.I., 2015, ‘Male partner’s role during pregnancy, labour and delivery: Expectations of pregnant women in Nigeria’, *International Journal of Health Sciences* 9(3), 305. 10.12816/0024697 26609295 PMC4633194

[CIT0002] Asefa, F., Geleto, A., Dessie, Y., 2014, ‘Male partners involvement in maternal ANC care: The view of women attending ANC in Harari public health institutions, eastern Ethiopia’, *Science Journal of Public Health* 2(3), 182–188. 10.11648/j.sjph.20140203.17

[CIT0003] Babalola, S. & Fatusi, A., 2009, ‘Determinants of use of maternal health services in Nigeria-looking beyond individual and household factors’, *BMC Pregnancy and Childbirth* 9, 91–13. 10.1186/1471-2393-9-43 PMC2754433 19754941

[CIT0004] Babbie, E. & Mouton, J., 2002, *Social research*, Wadsworth Group, Belmont, CA.

[CIT0005] Bayeh, E., 2016, ‘The role of empowering women and achieving gender equality to the sustainable development of Ethiopia’, *Pacific Science Review B: Humanities and Social Sciences* 2(1), 37–42.

[CIT0006] Davis, J., Vaughan, C., Nankinga, J., Davidson, L., Kigodi, H., Alalo, E. et al., 2018, ‘Expectant fathers’ participation in antenatal care services in Papua New Guinea: A qualitative inquiry’, *BMC Pregnancy and Childbirth* 18, 181–113. 10.1186/s12884-018-1759-4 29739351 PMC5941321

[CIT0007] Falade-Fatila, O. & Adebayo, A.M., 2020, ‘Male partners’ involvement in pregnancy related care among married men in Ibadan, Nigeria’, *Reproductive Health* 17, 1–12. 31992315 10.1186/s12978-020-0850-2 PMC6988334

[CIT0008] Forbes, F., Wynter, K., Wade, C., Zeleke, B.M., Fisher, J., 2018, ‘Male partner attendance at antenatal care and adherence to antenatal care guidelines: Secondary analysis of 2011 Ethiopian demographic and health survey data’, *BMC Pregnancy and Childbirth* 18(1), 1–11. 10.1186/s12884-018-1775-4 29743039 PMC5944090

[CIT0009] Galle, A., Cossa, H., Griffin, S., Osman, N., Roelens, K., Degomme, O., 2019, ‘Policymaker, health provider and community perspectives on male involvement during pregnancy in southern Mozambique: A qualitative study’, *BMC Pregnancy and Childbirth* 19(1), 1–14. 10.1186/s12884-019-2530-1 31660898 PMC6819364

[CIT0010] Ganle, J.K., Dery, I., Manu, A.A., Obeng, B., 2016, ‘If i go with him, i can’t talk with other women’: Understanding women’s resistance to, and acceptance of, men’s involvement in maternal and child healthcare in northern Ghana’, *Social Science & Medicine* 166, 195–204. 10.1016/j.socscimed.2016.08.030 27569661

[CIT0011] Guetterman, T.C., 2015, ‘Descriptions of sampling practices within five approaches to qualitative research in education and the health sciences’, *Forum Qualitative Sozialforschung/Forum: Qualitative Social Research* 16(2), 2–9.

[CIT0012] Guest, G., MacQueen, K.M. & Namey, E.E., 2011, *Applied thematic analysis*, Sage Publications, London.

[CIT0013] Kabagenyi, A., Jennings, L., Reid, A., Nalwadda, G., Ntozi, J., Atuyambe, L., 2014, ‘Barriers to male involvement in contraceptive uptake and reproductive health services: A qualitative study of men and women’s perceptions in two rural districts in Uganda’, *Reproductive Health* 11(1), 1–9. 24597502 10.1186/1742-4755-11-21 PMC3946591

[CIT0014] Kiptoo, S.J. & Kipmerewo, M., 2017, ‘Male partner involvement in antenatal care services in Mumias East and West sub-counties, Kakamega County, Kenya’, *Journal of Nursing and Health Science* 6(4), 37–46.

[CIT0015] Krueger, R.A. & Casey, M.A., 2015, ‘Focus group interviewing’, in K.E. Newcomer, H.P. Hatry & J.S. Wholey (eds.), *Handbook of practical program evaluation*, pp. 506–534, John Wiley & Sons, Inc., Hoboken, NJ.

[CIT0016] Kumbeni, M.T., Ziba, F.A., Alem, J.N. & Nborah, S.A., 2019, ‘Factors influencing male involvement in antenatal care in the Kassena Nankana Municipal in the Upper East Region, Ghana’, *European Scientific Journal* 15(21), 3.

[CIT0017] Kwambai, T.K., Dellicour, S., Desai, M., Ameh, C.A., Person, B., Achieng, F. et al., 2013, ‘Perspectives of men on antenatal and delivery care service utilisation in rural western Kenya: A qualitative study’, *BMC Pregnancy and Childbirth* 13(1), 1–10. 10.1186/1471-2393-13-134 23800139 PMC3691751

[CIT0018] Lincoln, Y.S. & Guba, E.G., 1985, *Naturalistic inquiry*, Sage, London.

[CIT0019] Maguire, M. & Delahunt, B., 2017, ‘Doing a thematic analysis: A practical, step-by-step guide for learning and teaching scholars’, *All Ireland Journal of Higher Education* 9(3), 1–2.

[CIT0020] Mathews, T., MacDorman, M.F. & Thoma, M.E., 2015, ‘Infant mortality statistics from the 2013 period linked birth/infant death data set’, *National Vital Statistics Reports* 64(9), 1–30. 26270610

[CIT0021] Matseke, M.G., Ruiter, R.A., Barylski, N., Rodriguez, V.J., Jones, D.L., Weiss, S.M., et al. 2017, ‘A qualitative exploration of the meaning and understanding of male partner involvement in pregnancy-related care among men in rural South Africa’, *Journal of Social, Behavioral and Health Sciences* 11, 1–4. PMC5945278 29755646

[CIT0022] Maluka, S.O. & Peneza, A.K., 2018, ‘Perceptions on male involvement in pregnancy and childbirth in Massai district, Tanzania: A qualitative study’, *Reproductive Health* 15, 68. 10.1186/s12978-018-0512-9 29678184 PMC5910565

[CIT0023] Mapunda, B., August, F., Mwakawanga, D., Mhando, I. & Mgaya, A., 2022, ‘Prevalence and barriers to male involvement in antenatal care in Dar es Salaam, Tanzania: A facility-based mixed-methods study’, *PloS One* 17(8), e0273316. 10.1371/journal.pone.0273316 35984819 PMC9390926

[CIT0024] Mersha, A.G., 2018, ‘Male involvement in the maternal health care system: Implication towards decreasing the high burden of maternal mortality’, *BMC Pregnancy and Childbirth* 18(1), 1–8. 10.1186/s12884-018-2139-9 30547771 PMC6295014

[CIT0025] Mohammed, B.H., Johnston, J.M., Vackova, D., Hassen, S.M., Yi, H., 2019, ‘The role of male partner in utilization of maternal health care services in Ethiopia: A community-based couple study’, *BMC Pregnancy and Childbirth* 19(1), 1–9. 10.1186/s12884-019-2176-z 30642280 PMC6332901

[CIT0026] Morgan, A.K., Awafo, B.A., Quartey, T. & Cobbold, J., 2022, ‘Husbands’ involvement in antenatal-related care in the Bosomtwe district of Ghana: Inquiry into the facilitators and barriers’, *Reproductive Health* 19(1), 1–14. 10.1186/s12978-022-01506-7 36456980 PMC9714231

[CIT0027] Morris, J.L., Short, S., Robson, L. & Andriatsihosena, M.S., 2014, ‘Maternal health practices, beliefs and traditions in southeast Madagascar’, *African Journal of Reproductive Health* 18(3), 101–117. 25438515

[CIT0028] Nesane, K., Maputle, S.M. & Shilubane, H. 2016, ‘Male partners’ views of involvement in maternal healthcare services at Makhado Municipality clinics, Limpopo Province, South Africa’, *African Journal of Primary Health Care and Family Medicine* 8(2), 1–5. 10.4102/phcfm.v8i2.929 PMC4887594 27380843

[CIT0029] Ongolly, F.K. & Bukachi, S.A., 2019, ‘Barriers to men’s involvement in antenatal and postnatal care in Betula, western Kenya’, *African Journal of Primary Health Care & Family Medicine* 11(1), 1–7. 10.4102/phcfm.v11i1.1911 PMC6676928 31368318

[CIT0030] Påfs, J., Musafili, A., Binder-Finnema, P., Klingberg-Allvin, M., Rulisa, S. & Essén, B., 2015, ‘They would never receive you without a husband’: Paradoxical barriers to antenatal care scale-up in Rwanda’, *Midwifery* 31(12), 1149–1156. 26471934 10.1016/j.midw.2015.09.010

[CIT0031] Rutberg, S. & Bouikidis, C.D., 2018, ‘Focusing on the fundamentals: A simplistic differentiation between qualitative and quantitative research’, *Nephrology Nursing Journal* 45(2), 209–213. 30303640

[CIT0032] Saah, F.I., Tarkang, E.E., Komesuor, J., Osei, E., Acquah, E. & Amu, H., 2019, ‘Involvement of male partners in skilled birth care in the North Dayi district, Ghana’, *International Journal of Reproductive Medicine* 2019, 2852861. 10.1155/2019/2852861 31355245 PMC6632491

[CIT0033] Selection, W.E.C.O.T., Medicines, U.O.E., Organization, W.H., 2014, *The selection and use of essential medicines: Report of the who expert committee, 2013 (including the 18th who model list of essential medicines and the 4th who model list of essential medicines for children)*, World Health Organization, viewed n.d., from https://iris.who.int/handle/10665/351172.

[CIT0034] Story, W.T., Barrington, C., Fordham, C., Sodzi-Tettey, S., Barker, P.M. & Singh, K., 2016, ‘Male involvement and accommodation during obstetric emergencies in rural Ghana: A qualitative analysis’, *International Perspectives on Sexual and Reproductive Health* 42(4), 211. 10.1363/42e2616 28825900 PMC6771418

[CIT0035] Suandi, D., Williams, P. & Bhattacharya, S., 2020, ‘Does involving male partners in antenatal care improve healthcare utilisation? Systematic review and meta-analysis of the published literature from low-and middle-income countries’, *International Health* 12(5), 484–498. 10.1093/inthealth/ihz073 31613327 PMC11701106

[CIT0036] Tessema, K.M., Mihirete, K.M., Mengesha, E.W., Nigussie, A.A. & Wondie, A.G., 2021, ‘The association between male involvement in institutional delivery and women’s use of institutional delivery in Debre Tabor town, north west Ethiopia: Community based survey’, *PloS one* 16(4), e0249917. 10.1371/journal.pone.0249917 33836011 PMC8034730

[CIT0037] Thapa, D.K. & Niehof, A., 2013, ‘Women’s autonomy and husbands’ involvement in maternal health care in Nepal’, *Social Science and Medicine* 93, 1–10. 10.1016/j.socscimed.2013.06.003 23906115

[CIT0038] Yaya, S., Uthman, O.A., Okonofua, F. & Bishwajit, G., 2019, ‘Decomposing the rural-urban gap in the factors of under-five mortality in sub-Saharan Africa? Evidence from 35 countries’, *BMC Public Health* 19(1), 1–10. 31113395 10.1186/s12889-019-6940-9 PMC6528236

[CIT0039] Yende, N., Van Rie, A., West, N.S., Bassett, J. & Schwartz, S.R., 2017, ‘Acceptability and preferences among men and women for male involvement in antenatal care’, *Journal of Pregnancy* 2017, 4758017. 10.1155/2017/4758017 28243473 PMC5294384

[CIT0040] Younas, M., Parpio, Y., Ali, T.S. & Awan, S., 2020, ‘Male partners’ knowledge and practices of antenatal care in district swat, Khyber Pakhtunkhwa, Pakistan: A cross-sectional study’, *Journal of Midwifery and Reproductive Health* 8(1), 2005.

[CIT0041] World Health Organization, 2015, *Strategies towards ending preventable maternal mortality*, WHO, Geneva.

